# The effect of nalbuphine on prevention of emergence delirium in children: a systematic review with meta-analysis^☆^

**DOI:** 10.1016/j.bjane.2024.844543

**Published:** 2024-07-22

**Authors:** Ka Ting Ng, Wei En Lim, Wan Yi Teoh, Mohd Fitry Bin Zainal Abidin

**Affiliations:** a University of Malaya, Department of Anesthesiology, Kuala Lumpur, Malaysia; b University of Glasgow, Department of Anesthesiology, Glasgow, United Kingdom; c University of Liverpool, Department of Medicine, Liverpool, United Kingdom

**Keywords:** Nalbuphine, Emergence delirium, Child, Pain, postoperative, Nausea, Vomiting

## Abstract

**Background:**

Emergence delirium remains a major postoperative concern for children undergoing surgery. Nalbuphine is a synthetic mixed agonist-antagonist opioid, which is believed to reduce the incidence of emergence delirium in children. The primary objective was to examine the effect of nalbuphine on emergence delirium in children undergoing surgery.

**Methods:**

Databases of MEDLINE, EMBASE, and CENTRAL were searched from their starting dates until April 2023. Randomized Clinical Trials (RCT) and observational studies comparing nalbuphine and control in children undergoing surgery were included.

**Results:**

Eight studies (n = 1466 patients) were eligible for inclusion of data analysis. Compared to the control, our pooled data showed that the nalbuphine group was associated with lower incidence of emergence delirium (RR = 0.38, 95% CI [0.30, 0.47], *p* < 0.001) and reduced postoperative pain scores (MD = -0.98, 95% CI [-1.92, -0.04], *p* = 0.04).

**Conclusions:**

This review showed the administration of nalbuphine is associated with significant decrease in the incidence of emergence delirium and postoperative pain scores among children undergoing surgery. However, due to limited sample size, high degree of heterogeneity and low level of evidence, future adequately powered trials are warranted to explore the efficacy of nalbuphine on emergence delirium among the pediatric population.

## Introduction

Emergence delirium is common in pediatric patients when they recover from anesthesia at the end of surgery, ranging from 10–80% depending on the type of anesthetic agent, criteria of emergence delirium and scoring system.^1^ It is characterized as a brief state of extreme irritability and dissociation with children who do not respond to consoling measures after anesthesia has been discontinued.^2^ This can lead to possible self-injury, such as children falling off the bed, removing intravenous/urinary catheters, and damaging surgical dressings.^3^ Furthermore, it is also associated with significant anxiety in parents, leading to poor parental satisfaction levels^4^ and prolonged hospital stays.^5^

Numerous studies have contributed to our understanding of prevention of emergence delirium via the administration of various drugs such as midazolam or propofol in children.^6^^,^^7^ However, their use is limited due to various adverse effects, such as bradycardia, hypotension, and slow recovery from anesthesia.^8^^,^^9^ These limitations warrant future studies of alternative drugs with better efficacy and safety profile.

Nalbuphine is a semi-synthetic opioid that acts as a κ-receptor agonist and µ-receptor antagonist and plays a crucial role in providing analgesic and sedation. Nalbuphine is equianalgesic to morphine with faster onset of action and has minimal adverse effects such as lower incidence of desaturation, nausea and vomiting.^10^ It also features a ceiling effect for respiratory depression, making it one of the safer analgesics for children.^11^ Current evidence shows that nalbuphine is used as an adjunctive anesthesia agent for pain management among pediatric patients.^12^^,^^13^ Several Randomized Controlled Trials (RCT) showed promising results on the efficacy of nalbuphine in lowering the incidence of emergence delirium in children as compared to placebo.^14^, ^15^, ^16^, ^17^, ^18^, ^19^ Nalbuphine reduces the incidence of emergence delirium, yet the underlying mechanism remains unclear.^14^

However, most of the established data on the efficacy of nalbuphine was performed on adults undergoing surgery.^20^^,^^21^ Therefore, a comprehensive systematic review and meta-analysis is warranted to summarize current evidence on the use of nalbuphine in children undergoing surgery.

We hypothesized that nalbuphine would attenuate the incidence of emergence delirium in children undergoing surgery. We also hypothesized that nalbuphine could reduce postoperative pain scores and the incidence of nausea and vomiting in children. The primary objective of this review was to examine the effect of nalbuphine on the incidence of emergence delirium in pediatric patients. Secondary objectives were to investigate the effect of nalbuphine on the postoperative pain score and incidence of nausea and vomiting in children after surgery.

## Methods

This review was carried out in accordance with the Cochrane Handbook of Systematic Reviews.^22^ Our study protocol was registered on PROSPERO, CRD42023416518 before the beginning of the systematic search for relevant articles. The research questions were formulated using a Population-Intervention-Comparison-Outcome (PICO) approach. The primary outcome was the incidence of emergence delirium among children undergoing surgery. Secondary outcomes included postoperative pain scores, incidence of nausea and vomiting, and incidence of desaturation.

### Literature search and study identification

Databases of the MEDLINE, EMBASE, and Cochrane Central Register of Trials were searched from their starting dates until April 2023. ClinicalTrials.gov.my and the WHO International Clinical Trial Registry were searched for any ongoing or unpublished clinical trials. No restrictions were applied based on the publication date or language of publication. The search terms and search strategy are listed in Supplementary Table 1. The inclusion criteria were listed below: 1) All Randomized Controlled Trials (RCTs) or observational studies comparing nalbuphine versus control groups were included; 2) All RCTs or observational studies comparing nalbuphine versus control groups involving children undergoing surgery, regardless of the type of surgery and reported outcomes.

Letters to editors, case reports, case series, and conference abstracts were excluded. Trials comparing nalbuphine and control in adult patients undergoing surgery were excluded. The references to all the included studies were searched for relevant articles that fulfilled our inclusion criteria. The authors of relevant studies were contacted at least three times if no response was received for any uncertainty in their data.

### Study selection and data extraction

The review was reported according to the guidelines of the Preferred Reporting Items for Systematic Review and Meta-Analyses Statement (PRISMA) 2020.^23^ Two authors (WT and WL) were briefed by the main author (KN) on the eligibility and exclusion criteria. Titles and abstracts of studies were systematically searched and identified based on the inclusion criteria by both review authors (WT and WL). The final selection of all included studies was discussed and agreed among all the three authors (KN, WT, and WL). Data from relevant studies were independently extracted by the two authors (WT and WL) using a standardized data collection form. Clinical characteristics of the included studies were documented by both authors (WT and WL) separately and cross-checked for any discrepancies, as illustrated in Table 1.

**Table 1:** Clinical characteristics of included studies.

Author	Year	References	Type of Surgery	Design	Drug	Dose of Nalbuphine	Control Group Drug	Dose of Control	Timing of Administration	Type of Anesthesia	Dose of Anesthesia	Mean Age (Control)^a^	Mean Age (Nalbuphine)^a^	Mean BMI (Control)^a^	Mean BMI (Nalbuphine)^a^	n^a^
Kim	2008	^18^	Strabismus Surgery	RCT	Nalbuphine	0.1 mg.kg^−1^	Normal Saline	0.1 mg.kg^−1^	End of Surgery	Sevoflurane	6 L.min^−1^	4.7 ± 1.3	4.8 ± 1.2	‒	‒	90
Chang	2008	^19^	Strabismus Surgery	RCT	Nalbuphine	0.2 mg.kg^−1^	Normal Saline	0.2 mg.kg^−1^	During Surgery except for high agitation score then nalbuphine is administered additionally after surgery	Desflurane	4%−6% with N_2_O:O_2_ is 2:1	7.3 ± 2.3	7.0 ± 3.3	‒	‒	41
Mohammed	2017	^16^	Tonsillectomy with or without adenoidectomy	RCT	Nalbuphine	0.1 mg.kg^−1^	Midazolam	0.03 mg.kg^−1^	5 min before the end of surgery	Sevoflurane	6 L.min^−1^ with increments of 1% at each breath up to 8%.	5.7±1.3	5.6±1.3	‒	‒	90
Vavrina	2017	^17^	Adenotonsillectomy	Observational Study	Nalbuphine	0.17 ± 0.03 mg.kg^−1^	Alfentanil	0.012 ± 0.004 mg.kg^−1^	During surgery	Propofol or 40% Oxygen, 60% Nitrous Oxide and Sevoflurane	‒	5.7 ± 3.1	5.1 ± 1.7	‒	‒	114
Zhao	2017	^14^	Dental Surgery	RCT	Nalbuphine	0.1 mg.kg^−1^	Normal Saline	0.1 mg.kg^−1^	Before the end of surgery	Sevoflurane	8% in 100% oxygen	4.4 ± 1.2	4.5 ± 1.3	‒	‒	84
El-Din	2018	^15^	Adenotonsillectomy	RCT	Nalbuphine	0.1 mg.kg^−1^	Midazolam	0.03 mg.kg^−1^	5 min before the end of surgery	Sevoflurane	6 L.min^−1^ with increments of 1% at each breath up to 8%	5.7 ± 1.3	5.6 ± 1.3	‒	‒	90
Elagamy	2020	^30^	Adenotonsillectomy	RCT	Nalbuphine	0.1 mg.kg^−1^	Dexmedetomidine	0.5 µg.kg^−1^	During the surgery	Sevoflurane	8% in 100% of oxygen	4.5 ± 0.8	4.7 ± 1	-	-	160
He	2023	^14^	Adenotonsillectomy	RCT	Nalbuphine	0.1 mg.kg^−1^	Normal Saline	0.1 mg.kg^−1^	During surgery	Sevoflurane	6 L.min^−1^	5.1 ± 1.8	5.0 ± 1.9	15.7 ± 2.5	15.5 ± 2.1	797

a Values are in Mean ± Standard Deviation. RCT, Randomized Controlled Trial; BMI, Body Mass Index; n, Total Sample Size.

### Risk of bias assessment

All the included RCTs were evaluated for the risk of bias using the Cochrane Collaboration Risk of Bias Assessment Tool by two authors (WT and WL) independently.^24^ The observational studies were assessed for the risk of bias using the Newcastle-Ottawa Scale.^25^ The principles of the Grading of Recommendations, Assessment, Development, and Evaluations (GRADE) system were applied to assess the quality of evidence of the primary and secondary outcomes.^26^ The summary of findings and the level of evidence were carried out independently by both authors (WT and WL) using the GRADEpro/GDT software (Guideline Development Tool [Software]). The certainty of evidence was assessed based on the five major criteria (risk of bias, inconsistency, indirectness, imprecision, and publication bias).^24^ Any disagreements were solved by the main author (KN).

### Summary measures and synthesis of results

Statistical meta-analysis was conducted using Review Manager version 5.4 (The Cochrane Collaboration, Copenhagen, Denmark).^27^ A two-tail *p*-value < 0.05 was indicated as having statistical significance. Our findings were reported as Risk Ratio (RR) and 95% Confidence Interval for binary outcomes. With regards to the continuous outcomes, the Mean Difference (MD) and 95% Confidence Interval were calculated. Due to the variation in clinical settings, the degree of heterogeneity of the pooled data was evaluated. The I-square (I^2^) with values of < 40%, 40% to 60%, and > 60% were classified as low, moderate, and high, respectively. A fixed-effects model was applied to summarize the estimates of outcomes. If significant heterogeneity (I^2^ > 60%) was observed, a random-effects model was utilized to analyze the data. When the values were reported as median or interquartile range, these values were converted to mean and standard deviation.^28^ Sensitivity analysis was performed on the primary outcome by focusing on the RCT studies only to eliminate the introduction of bias from the inclusion of observational studies. However, funnel plot analysis to test for publication bias could not be conducted due to the limited number of studies (number of studies ≤ 10).

## Results

**Figure 1:**
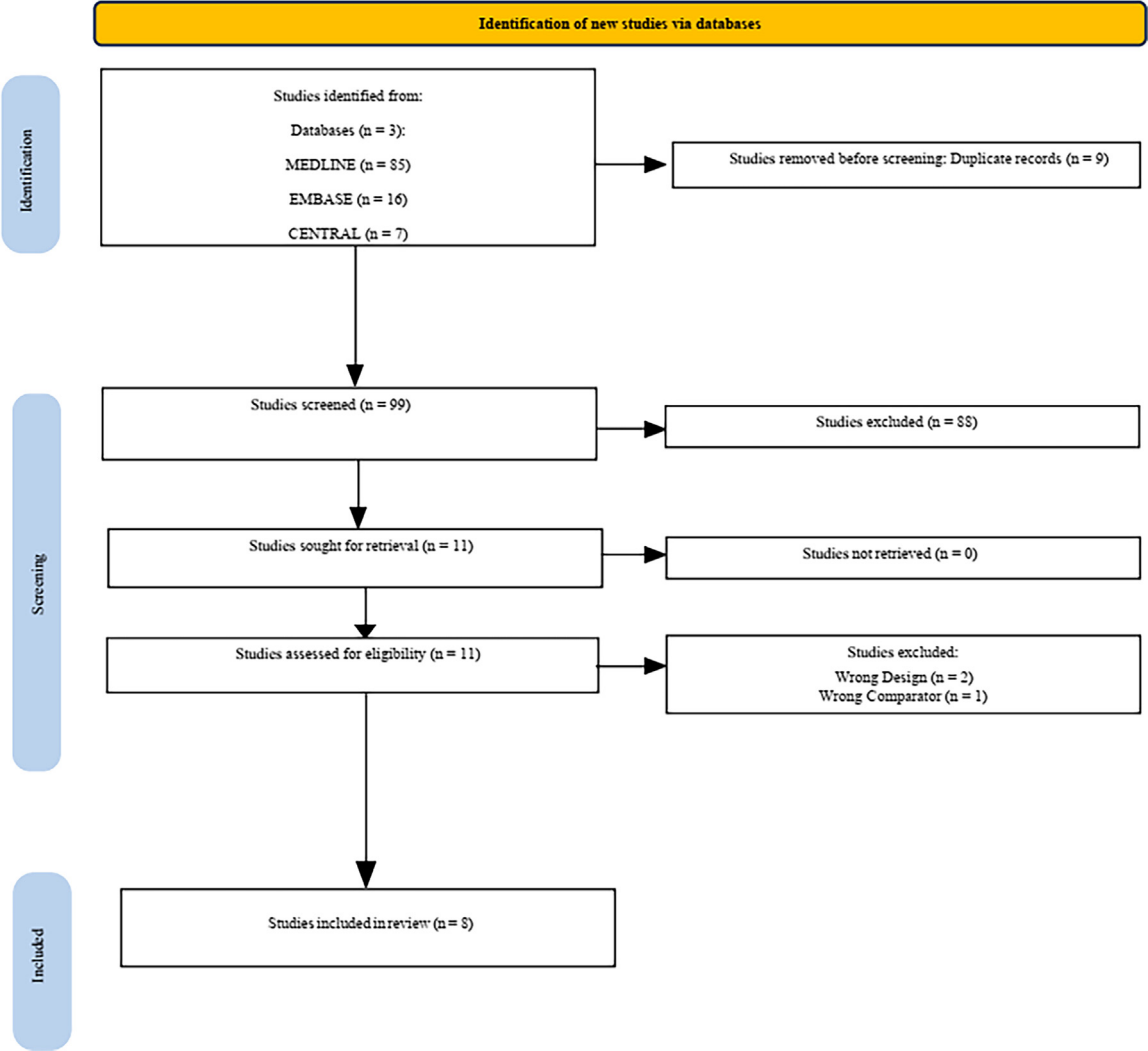
Prisma diagram of systematic review.

The study selection process is illustrated in the PRISMA diagram (Fig. 1). Our search generated 99 non-duplicated articles for title and abstract screening. Of all, eleven articles were identified and retrieved for full text screening. After applying the inclusion and exclusion criteria, three articles were excluded, as shown in Supplementary Table 2. A total of eight studies (seven RCTs, one observational study; n = 1466) were included in the quantitative analysis of the primary outcome of the review, whereas one of studies was not included owing to its type of data being continuous instead of dichotomous.^29^ The search of the main registries identified two ongoing studies (Supplementary Table 3).

The clinical characteristics of all the included studies are depicted in Table 1. Among all the included studies, four were adenotonsillectomy,^14^^,^^17^^,^^18^^,^^29^ two were strabismus surgery,^15^^,^^30^ one was dental surgery^19^ and another study was tonsillectomy with or without adenoidectomy.^16^ The dose of nalbuphine ranged from 0.1 mg.kg^−1^ to as high as 0.2 mg.kg^−1^. With regards to the control group, two studies used midazolam,^16^^,^^17^ one gave alfentanil,^18^ one used dexmedetomidine^29^ while the remaining four studies used normal saline as a placebo.^14^^,^^15^^,^^19^^,^^30^ All the included studies administered sevoflurane for maintenance of anesthesia,^14^, ^15^, ^16^^,^^18^^,^^19^^,^^29^ except for one of the studies that used desflurane^30^ in pediatric patients. The average ages of the nalbuphine and control group were 5.3 years old (Standard Deviation [SD = 1.45]) and 5.4 yearsold (SD = 1.63), respectively. The publication year of the included studies ranged from 2008 to 2023. The data analysis of the primary and secondary outcomes is shown in Table 2 whereas the level of evidence is illustrated in Table 3. In the overall risk of bias assessment, two studies were of low risk of bias^14^^,^^29^ and six studies were evaluated as unclear or high risk of bias.^15^, ^16^, ^17^, ^18^, ^19^^,^^30^ because of the lack of blinding of participants or personnel, and the lack of blinding of outcome assessors (Supplementary Table 4). The PRISMA Checklist was done according to the PRISMA 2020 guidelines.^23^

**Table 2:** Summary of findings of primary and secondary outcomes.

N°	Outcomes	Trials	n	I^2^(%)	MD/RR (95% CI)	p-value
1	Incidence of Emergence Delirium (Main Analysis)	7	1,319	0	RR: 0.38 [0.30, 0.47]	<0.001
2	Incidence of Emergence Delirium					
	a) Aono's Scale	3	264	0	0.35 [0.22, 0.56]	<0.001
	b) PAED Score	2	881	0	0.37 [0.28, 0.50]	<0.001
	c) Watcha Scale	1	114	N/A	0.50 [0.27, 0.92]	0.03
	d) Cole Agitation Score	1	60	N/A	0.37 [0.18, 0.74]	0.005
	e) Placebo	4	1,025	0	0.37 [0.29, 0.48]	<0.001
	f) Control	3	294	0	0.40 [0.26, 0.63]	<0.001
3	Pain Score (Main Analysis)	3	971	68	MD = −0.98 [−1.92, −0.04]	0.04
4	Pain Score					
	a) CHEOPS	2	174	0	−1.52 [−2.40, −0.63]	<0.001
	b) FLACC	1	797	N/A	−0.40 [−0.60, −0.20]	<0.001
5	Incidence of Nausea and Vomiting	5	1,191	0	RR = 1.45 [0.78, 2.70]	0.24
6	Incidence of Desaturation	3	234	N/A	RR = 5.00 [0.25, 99.95]	0.29

n, Total Sample Size; MD, Mean Difference; RR, Risk Ratio; CI, Confidence Interval; I^2^, Heterogeneity; N/A, Not Applicable; CHEOPS, The Children's Hospital of Eastern Ontario Pain Scale; FLACC, The Face, Legs, Activity, Cry, Consolability Scale; PAED, Pediatric Anesthesia Emergence Delirium.

**Table 3:** Level of Evidence. The level of evidence ranged from very low to low.

Question: Nalbuphine compared to Control for the incidence of emergence delirium.											
Certainty assessment							№ of patients		Effect		Certainty
№ of studies	Study design	Risk of bias	Inconsistency	Indirectness	Imprecision	Other considerations	Nalbuphine	Control	Relative (95% CI)	Absolute (95% CI)	
**Incidence of Emergence Delirium**											
7	Randomised trials	Very serious^a^	Not serious	Not serious	Serious^b^	Publication bias strongly suspected^c^	86/659 (13.1%)	228/660 (34.5%)	**RR 0.38** (0.30 to 0.47)	**214 fewer per 1,000** (from 242 fewer to 183 fewer)	⨁○○○ Very low
**Pain Scale**											
3	Randomised trials	Not serious	Not serious	Not serious	Serious^b^	Publication bias strongly suspected^c^	486	485	-	MD 0.98 **lower** (1.92 lower to 0.04 lower)	⨁⨁○○ Low
**Incidence of Nausea and Vomiting**											
5	Randomised trials	Serious^d^	Not serious	Not serious	Serious^b^	Publication bias strongly suspected^c^	22/617 (3.6%)	15/615 (2.4%)	**RR 1.45** (0.78 to 2.70)	**11 more per 1,000** (from 5 fewer to 41 more)	⨁○○○ Very low
**Incidence of Desaturation**											
3	Randomised trials	Serious^e^	Not serious	Not serious	Serious^b^	Publication bias strongly suspected^c^	2/117 (1.7%)	0/117 (0.0%)	**RR 5.00** (0.25 to 99.95)	**0 fewer per 1,000** (from 0 fewer to 0 fewer)	⨁○○○ Very low

CI, Confidence Interval; MD, Mean Difference; RR, Risk Ratio.

Explanations:

a One of the included studies is an observational study.

b Sample group size < 400.

c Small studies with positive results.

d Half of the included studies possess high or unclear risk of bias.

e All of the studies possess high or unclear risk of bias.

### Primary outcome: incidence of emergence delirium

**Figure 2:**
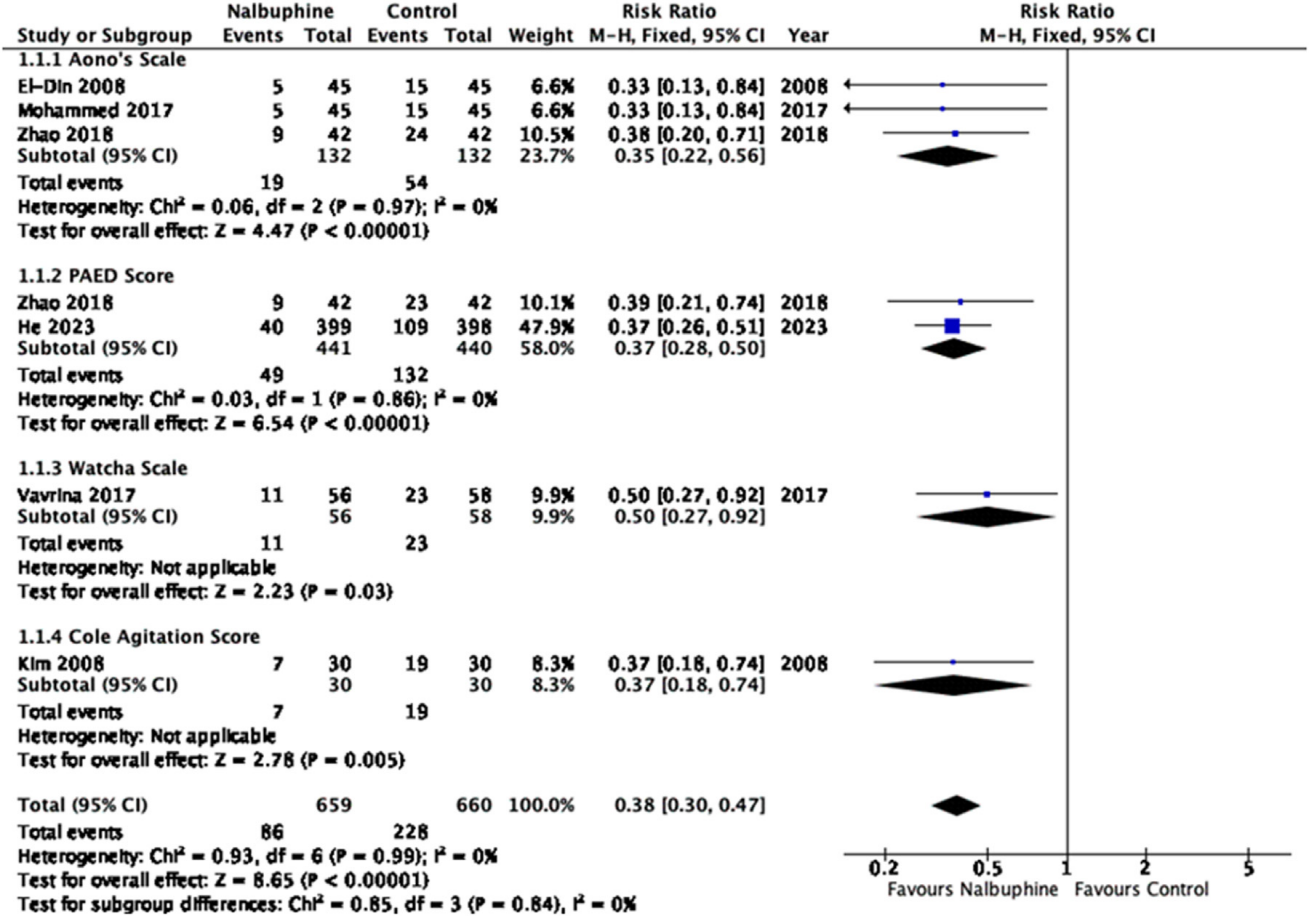
Incidence of Emergence Delirium in pediatric patients receiving nalbuphine or control drugs. Nalbuphine significantly reduced the incidence of emergence delirium in comparison to the control group.

In our primary analysis, six individual trials were identified. Within these trials, a total of seven distinct comparison groups were examined. Six trials with seven sets of data (n = 1319 patients; nalbuphine group = 659, control group = 660) investigated the effects of nalbuphine versus control on the incidence of emergence delirium.^14^, ^15^, ^16^^,^^18^^,^^19^ The incidence of emergence delirium in the nalbuphine and control groups was 13.05% and 34.65% respectively, which was statistically significant (RR = 0.38, 95% CI [0.30, 0.47], *p* < 0.001, level of evidence: very low) (Fig. 2). The statistical heterogeneity across studies was low (I^2^ = 0%). Subgroup analysis was performed based on different types of emergence delirium scoring systems, specifically Aono's scale (n = 264, RR = 0.35, 95% CI [0.22, 0.56], *p* < 0.001), Pediatric Anesthesia Emergence Delirium (PAED) Score (n = 881, RR = 0.37, 95% CI [0.28, 0.50], *p* < 0.001, I^2^ = 0%), Watcha Scale (n = 114, RR = 0.50, 95% CI [0.27, 0.92], *p* = 0.03) and Cole Agitation Score (n = 60, RR = 0.37, 95% CI [0.18,0.74], *p* = 0.005) (Fig. 2). Another subgroup analysis was performed on the use of nalbuphine versus placebo (n = 1,025, RR = 0.37, 95% CI [0.29, 0.48], p < 0.001) and control (n = 294, RR = 0.40, 95% CI [0.26, 0.63], *p* < 0.001) (Supplementary Fig. 1). Sensitivity analysis of RCTs only showed that the nalbuphine group was associated with a lower incidence of emergence delirium in children undergoing surgery (n = 1,205, RR = 0.37, 95% CI [0.29, 0.46], *p* < 0.001) (Supplementary Fig. 2).

### Secondary outcomes: pain scores, incidence of nausea and vomiting

In comparison to the control group, the nalbuphine group was associated with statistically lower postoperative pain scores (nalbuphine group = 486, control group = 485, MD = -0.98, 95% CI [-1.92, -0.04], *p* = 0.04, level of evidence: low) (Supplementary Fig. 3).^14^^,^^17^^,^^19^ However, a high degree of heterogeneity was noted across studies (I^2^ = 68%). Subgroup analysis based on different scoring systems such as FLACC (Face, Legs, Activity, Cry, Consolability Scale) (MD = -0.40, 95% CI [-0.60, -0.20], *p* < 0.001) and Children's Hospital of Eastern Ontario Pain Scale (CHEOPS) systems (MD = -1.52, 95% CI [-2.40, -0.63], *p* < 0.001) revealed that postoperative pain scores were statistically lower in the nalbuphine group than in the control group (Supplementary Fig. 3).^14^^,^^17^^,^^19^ In terms of post-operative nausea and vomiting, the pooled data showed no significant differences between the nalbuphine and control groups (n = 1,164, nalbuphine group = 596, control group = 595, RR = 1.45, 95% CI [0.78, 2.70], *p* = 0.24, I^2^ = 0%, level of evidence: very low) (Supplementary Fig. 5).^14^^,^^15^^,^^17^^,^^19^^,^^29^

## Discussion

Our review demonstrated that nalbuphine was associated with decreased incidence of emergence delirium and reduced postoperative pain scores in children undergoing surgery. However, no significant difference was noted in the incidence of postoperative nausea and vomiting. The level of evidence ranged from very low to low due to risk of bias, imprecision, and strong suspicion of publication bias.

Although the mechanism of action of nalbuphine on emergence delirium remains unclear, our pooled data showed that children undergoing surgery randomized to nalbuphine were associated with a lower incidence of emergence delirium and lower postoperative pain scores. Nalbuphine is a mixed opioid agonist-antagonist that has agonist action on the kappa-receptor found in the brain and spinal cord to produce analgesia and sedative effects^31^ which can be reversed by naloxone.^11^ Its antagonist action on the μ-opioid receptor produces a capping effect on respiratory depression, and the respiratory depression will not worsen with any further increment of dosage.^32^ This ceiling effect is crucial in preventing severe respiratory depression, making nalbuphine a safer drug for treating pediatric patients. However, there are other confounding factors, such as the type of short-acting volatile anesthesia, type of surgery, and patient anxiety, that could influence the occurrence of emergence delirium.^33^

One of the hypotheses by Lewis on the development of emergence delirium asserts that the lack of clearance of volatile anesthetics can disrupt the balance of excitation and inhibition on the central nervous system, leading to different recovery rates of brain function after surgery.^34^ In addition, these inhalational agents like desflurane and sevoflurane possess a low blood/gas partition coefficient (0.42 and 0.69 respectively) as compared to isoflurane^35^ which may cause rapid awakening from anesthesia, leading to dissociation and altered cognition in children. Most of the included studies applied either sevoflurane or desflurane to maintain anesthesia in pediatric patients whereas one of the studies utilized propofol, implying that the impact of nalbuphine on emergence delirium cannot be fully elucidated in children who received either total intravenous anesthesia or inhalational anesthesia. Recent findings by Voepel-Lewis and colleagues reported that Ear, Nose, and Throat (ENT) surgery (55.4%) is an independent risk factor that can contribute to the incidence of emergence delirium among pediatric patients.^34^ Therefore, the inclusion of ENT surgery and other types of surgeries in our review may convey a high degree of variability in our pooled analysis, making the interpretation of the impact of nalbuphine on emergence delirium difficult due to limited data available in the literature.

The incidence of emergence delirium was diagnosed based on different scoring systems (PAED score, Watcha scale) in all the included studies, which could have introduced bias to our primary outcome. For example, the Watcha Scale, Aono's Scale, and Cole Agitation Score are measured based on crying and inconsolability only, which may cause Type II error due to the lower sensitivity of 0.34 and specificity of 0.95.^36^ In contrast, the PAED scale incorporates a wider range of behavior such as awareness of surroundings and making eye contact with a higher sensitivity of 0.93 and specificity of 0.94.^36^ Another important aspect was the different cut-off value of the PAED scores for emergence delirium in our included studies; for instance, He and colleagues indicated a cut-off ≥ 12 on the PAED scale to diagnose the incidence of emergence delirium^14^ while Zhao used a threshold value ≥10.^19^ Therefore, the assessment tool of emergence delirium and the specific cut-off point should be standardized in future RCTs to eliminate this assessment bias.^6^^,^^37^

It is believed that nalbuphine reduces the release of pro-inflammatory cytokines and oxidant stress factors, such as IL-6, tumor necrosis factor-alpha and Malondialdehyde (MDA), which exert good analgesia control to patients undergoing surgery.^38^ Ruan and colleagues concluded that nalbuphine down-regulates the nuclear factor of kappa-light chain enhancer of the activated B-cells, which helped to reduce the amount of visceral pain in rat models.^39^ In addition, nalbuphine is demonstrated to block central sensitization of pain,^40^ which is defined as the state of increased neuronal excitability and recruitment of non-nociceptive A-β fibers into the nociceptive pathway.^41^ Our finding was consistent with the results of previous experimental studies, demonstrating the beneficial analgesic effect of nalbuphine on postoperative pain relief in children. Similar conclusions were drawn by Kruszynski and colleagues showing that nalbuphine could be a better alternative to morphine in preventing postoperative pain due to its longer duration of action (4–5 hours).^42^

Measurement of pain scores was standardized in most of the included studies using the Children's Hospital of Eastern Ontario Pain Scale (CHEOPS), which is the one of the most appropriate tools for monitoring postoperative pain among children with high sensitivity of 0.93 and high specificity of 0.67.^43^ However, when given the same type of pain stimuli, pain scores can differ among patients depending on the individual's tolerance to pain and the use of other adjuncts analgesic medications.^44^ In a study by He,^14^ the synergic effect with nalbuphine and sufentanil/remifentanil may have provided better postoperative pain control in children after surgery.^45^ In a sensitivity analysis by removing this study, the mean difference of the postoperative pain scale remained statistically significant with a lower degree of heterogeneity. However, our finding might be premature due to the small sample size of included studies.

Nalbuphine exerts antagonist activity on µ receptors, which is theorized to lower the incidence of nausea and vomiting as compared to other opioids that have full agonist activity on the µ receptors.^46^ A meta-analysis of 15 trials (820 adult patients) showed a lower incidence of vomiting and nausea in the nalbuphine group as compared to the morphine group.^47^ Our review disagreed with the previous meta-analysis as our pooled data of a small sample size revealed no significant difference in the incidence of postoperative nausea and vomiting in children undergoing surgery. The administration of opioids activates µ receptors, leading to centrally mediated decrease in respiratory rate, which could cause desaturation^48^ and hypoxic insults to patients after surgery.^49^ It is believed that nalbuphine's antagonistic activity on µ receptors can minimize the risk of respiratory depression.^11^ Our review revealed no significant differences between the nalbuphine and control groups on the incidence of desaturation. However, given the limited number of studies with small sample size, these findings must be interpreted with caution. Therefore, the effects of nalbuphine on postoperative nausea and vomiting and incidence of desaturation remain to be elucidated in future adequately powered RCTs.

Our systematic review has several limitations. Firstly, one of the limitations is contributed by the lack of a standardized definition of emergence delirium across all the included studies. Besides, most of the included studies were underpowered, with each arm possessing less than 400 patients. In addition, a significant portion of the sample size was contributed by a single study (He et al), which accounted for almost 50% of the meta-analysis's weight, which may have skewed the overall results. Most of the included studies were underpowered to study our primary outcome. To date, there are limited studies on the use of nalbuphine to prevent emergence delirium. This could be due to concerns regarding the safety and efficacy profile of nalbuphine and its potential interactions with other anesthesia drugs, specifically among the pediatric population. Therefore, future adequately powered RCTs are warranted to study the true impact of nalbuphine on emergence delirium among children undergoing surgery.

In conclusion, to the best of our knowledge, this was the first systematic review and meta-analysis exploring the use of nalbuphine in the incidence of emergence delirium in children undergoing surgery. This meta-analysis demonstrated the promising effects of nalbuphine in lowering the incidence of emergence delirium in pediatric patients. However, small sample size and low quality of evidence limit a strong recommendation of the use of nalbuphine on emergence delirium in children.

## Conflicts of interest

The authors declare no conflicts of interest.

## Appendix Supplementary materials

Image, application 1
